# Evaluation of the transmissibility of norovirus and the effectiveness of prevention and control measures for schools in Jiangsu Province

**DOI:** 10.1080/07853890.2023.2246474

**Published:** 2023-08-21

**Authors:** Jing Wang, Jia Rui, Yuanzhao Zhu, Xiaohao Guo, Buasiyamu Abudunaibi, Benhua Zhao, Yanhua Su, Tianmu Chen, Jianli Hu

**Affiliations:** a State Key Laboratory of Vaccines for Infectious Diseases, Xiang An Biomedicine Laboratory, School of Public Health, Xiamen University, Xiamen City, Fujian Province, People’s Republic of China; b Nanjing Center for Disease Control and Prevention, Nanjing, Jiangsu Province, People’s Republic of China; c Jiangsu Provincial Center for Disease Control and Prevention, Nanjing, Jiangsu Province, People’s Republic of China

**Keywords:** Norovirus, transmissibility, total attack rate, peak incidence

## Abstract

**Objective:**

This study aims to estimate the transmissibility of norovirus outbreaks in schools by different transmission routes, and to evaluate the effects of isolation, school-closure and disinfection measures under different intervention intensities, finally, scientific prevention and control suggestions are proposed.

**Method:**

23 outbreaks of norovirus infectious diarrhea occurring in Jiangsu Province’s school from 2012-2018 were selected and fitted to the model. The data includes various types of school places and pathogen genotype. A ‘SEIAQRW’ model with two transmission routes was established. The transmissibility of each outbreak was assessed using effective reproduction number, the efficacy of different intervention measures and intensities were evaluated by calculating the total attack rate and peak incidence.

**Results:**

The mean effective reproduction number of noroviruses was estimated to be 8.92 for the human-to-human route of transmission and 2.19 for the water or food-to-human route of transmission. When all symptomatic cases were isolated, the median peak incidence for both transmission routes both being less than 1.8%. There was a smaller reduction in total attack rate compared to peak incidence, the median total attack rate for the two transmission routes decreased by 17.59% and 42.09%, respectively. When the effect of school-closure or disinfection is more than 90%, the total attack rate and peak incidence in the human-to-human route are reduced by more than 90% compared to no intervention, and the peak incidence in the water or food-to-human routes can be reduced to less than 1.4%, but the reduction in the total attack rate is only 50% or so.

**Conclusion:**

Norovirus outbreaks have a high rate of transmission in schools. In the case of norovirus outbreaks, isolation should be complemented by other interventions, and the implementation of high-intensity school closures or disinfection of the external environment can be effective in reducing the spread of the virus.

## Introduction

Norovirus infectious diarrhea has attracted increasing attention in recent years. Globally, norovirus is one of the common causes of acute gastroenteritis, and the prevalence of norovirus in acute gastroenteritis cases is approximately 20% [1,2]. Norovirus [3,4], being a mutable RNA virus, does not provide long-term immunity after infection in the population, and there is not effective vaccine against norovirus [5]. In addition, researches [6–8] have showed that norovirus has a high viral excretion capacity and can cause disease at low doses. Norovirus can survive in various environments and are able to transmit from person to person in various ways, such as direct interpersonal contact, ingestion of contaminated water or food, or exposure to contaminated environments. Therefore, once norovirus is present, it can easily cause transmission if not prevented and controlled in a timely manner.

Studies [9–11] have shown that outbreaks of norovirus have occurred in many provinces of China, mainly in school settings, which greatly affects the stability of education and the health of students. Several transmission dynamics models have been applied to study the transmissibility of norovirus outbreaks in schools and to assess the effectiveness of interventions, such as Xu Yucheng [12] used the SEIR model to evaluated the intervention effect of school-closure and disinfection measures for norovirus outbreaks in Shenzhen school premises. Chen Tianmu [13–15] further studied on this basis and further divided infected individuals into symptomatic and asymptomatic individuals and then constructed the SEIAR model (susceptible-exposed-symptomatic-asymptomatic-recovered), and then evaluated the transmissibility of norovirus and the prevention and control effects of interventions. Most studies focused on the human-to-human transmission route, with only a few of them considered a second transmission route, and yet, did not evaluate the effectiveness of corresponding interventions.

In this study, 23 norovirus outbreaks occurring in separate schools in Jiangsu Province from 2012 to 2018 were selected to assess the transmissibility of norovirus outbreaks in schools for each transmission route, which was fitted in the SEIAQRW dynamics model. The effectiveness of three interventions, namely, isolation, school closure and disinfection with different intervention intensities were quantitatively studied, providing a theoretical basis for rational selection and a combination of prevention and control measures.

## Methods

### Research subjects

Data on norovirus outbreaks in Jiangsu Province from 2012 to 2018 were categorized [15], including date of onset, number of cases, virus genotype, and place of occurrence, which allowed for the calculation of transmissibility. And the data used for this analysis were previously collected as routine outbreak surveillance and this was a retrospective analysis. Norovirus outbreaks with a clear genotype and occurred in schools were screened. Then, taking the transmission route as the main classification basis, the norovirus outbreaks in various types of places involved in different virus genotypes (based on VP1 sequences) were selected as the study objects, and the school sites were divided into five categories, which were kindergarten, primary school, middle school, Common Colleges and Secondary vocational school, nine-year school and twelve-year school. Finally, a total of 23 norovirus outbreaks were obtained, including 19 human-to-human outbreaks involving ten virus genotypes in five categories, and 4 water or food-to-human outbreaks involving four virus genotypes in three categories.

### Construct a model for the spread of the norovirus outbreak

**Figure 1.**
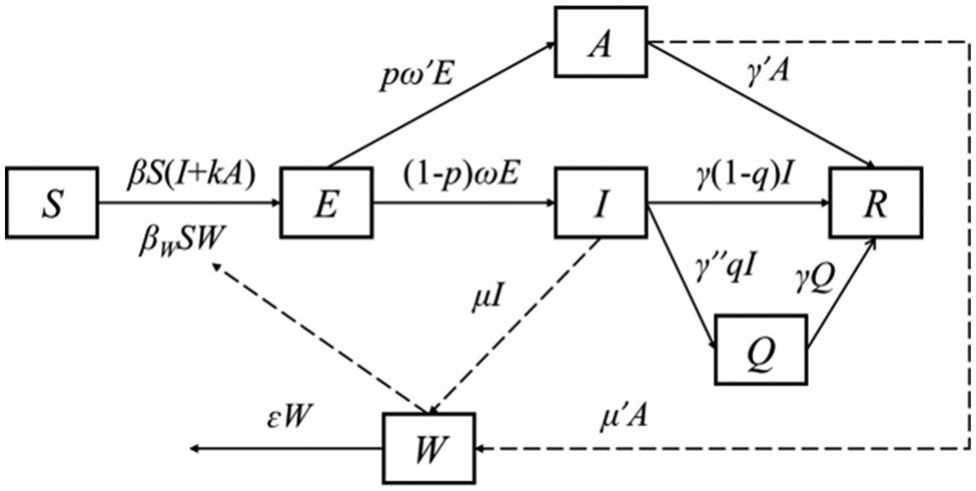
Flowchart of the SEIAQRW model for norovirus outbreak.

The SEIAQRW model was constructed by combining the disease characteristics of norovirus, two transmission routes and the implementation of prevention and control measures (Figure 1).

The model partitions the whole population into six sub-groups: susceptible (*S*), exposed (*E*), symptomatic (*I*), asymptomatic (*A*), isolated (*Q*), recovered (*R*), and contaminated water or food transmission medium (*W*). The system of differential equations is given as follows:

$$\frac{dS}{dt}=-\beta S(I+kA)-\beta_{w}SW$$

$$\frac{dE}{dt}=\beta S(I+kA)+\beta_{w}SW-p\omega^{\prime }E-(1-p)\omega E$$

$$\frac{dI}{dt}=(1-p)\omega E-\gamma (1-q)I-{\gamma^{\prime }}^{\prime }qI$$

$$\frac{dA}{dt}=p\omega^{\prime }E-\gamma^{\prime }A$$

$$\frac{dQ}{dt}={\gamma^{\prime }}^{\prime }qI-\gamma Q$$

$$\frac{dR}{dt}=\gamma (1-q)I+\gamma^{\prime }A+\gamma Q$$

$$\frac{dW}{dt}=\mu I+\mu^{\prime }A-\varepsilon W$$

$$\mu^{\prime }=c\mu$$

In the above equations, derivatives dS/dt, dE/dt, dI/dt, dA/dt, dQ/dt, dR/dt represent the rates of changes in corresponding population size at *t*-time, and dW/dt is the rate of change in virus excretion and extinction in the environment at *t*-time. The definition and value of each parameter are specified in the parameter estimation section.

### A SEIAQRW model without interventions

Without interventions, all parameters related to the variables Q and Q in the SEIAQRW model were set to 0. Since the variable W has practical significance only when the transmission routes includes water/food-to-human route, we used a further degenerated SEIAR model for scenarios where norovirus transmitted only through the human-to-human contact, by setting the variable W and parameters *β_W_*, *μ*, *μ′* and *ɛ* as 0 (Figure 1).

### A SEIAQRW model for isolation measure implemented

In norovirus outbreaks, isolation measures were applied only for symptomatic I, since asymptomatic A was more difficult to detect. It was assumed that when a symptomatic I was detected, the authorities would take isolation measures within a isolation efficiency *γ″* = 0.5 d, and people in Q would recover at the same rate as people in I. When isolation measures were taken, the proportion of I entering Q changed with isolation efficiency *γ″*, and the rest of the population changed as it would without intervention (Figure 1).

### A SEIAQRW model for school-closure measure implemented

When schools closed, the transmission rate coefficient *β* in the model decreased as the average number of people each person contacts per day decreased. The average number of personal contacts for each people per day after school-closure was the product of the average number of personal contacts for each people per day(*m*)and the coefficient of the average number of personal contacts for each people per day after school-closure(*h*). There are two formulas for calculating *β* after school-closure: if the *β* of norovirus outbreak fitted in the state of no intervention is less than 1, after school closure, $\beta =1-{\left(1-\mathrm{e}\right)}^{mh}$, otherwise, β=mhⅇ, and e is the infection probability when exposure to the virus once. Other parameters and initial values remain unchanged (Figure 1).

### A SEIAQRW model for disinfection measure implemented

If the external environment disinfection measure is taken for the norovirus outbreaks, such as remove vomit, contaminated water or food, or spray chlorine-containing disinfectants into classrooms and toilets, the transmission rate coefficient *β* will decrease due to the reduction of probability of infection *via* a single contact with the contagious environment. There are two formulas for calculating *β* after school-closure: if the *β* of norovirus outbreak fitted in the state of no intervention is less than 1, after school suspension, $\beta =1-{\left(1-x\mathrm{e}\right)}^{m}$, otherwise, β=mxⅇ, and x is the coefficient of infection probability when exposure to the virus once after disinfection. In addition, because the infectious dose of norovirus is extremely low, once the water or food is contagious, whether different individuals would be infected depends mainly on whether there is effective contact with contagious water or food, so the study hypothesizes that *β_W_* is 0 when *x* = 0 after disinfection, and *β_W_* is unchanged in the other cases. The rest of the population remain unchanged as it was in the scenario without intervention (Figure 1).

### Parameter estimation

A total of 21 parameters were involved in this study, their definition, unit, values and sources are shown in Table 1.

**Table 1.** Model parameter definitions and valuation methods.

Parameter	Definition	Value	Unit	Parameter source
*B*	Human-to-human transmit rate coefficient		km^2^·人^−1^·day^−1^	Model fitting
*β_W_*	Water or food-to-human transmit rate coefficient		mL^3^·virus^−1^·day^−1^	Model fitting
*K*	Relative transmissibility of asymptomatic individuals versus the symptomatic	0.05	1	Literature [16]
*W*	Inverse of incubation period	1	day^−1^	Literature [17,18]
*w′*	Inverse of latent period	1	day^−1^	Literature [19]
*P*	The ratio of the asymptomatic people	0.3	1	Literature [19,20,21]
*Γ*	Recovery rate for symptomatic people	0.3333	day^−1^	Literature [22–26]
*γ′*	Recovery rate for asymptomatic people	0.03846	day^−1^	Literature [19]
*γ″*	Isolation rate of symptomatic people	2	day^−1^	Assumption
*μ*	Virus excretion coefficient of the symptomatic people	——	virus^−1^·mL^−3^·day^−1^ ·km^2^·person^−1^	*c*/*μ*
*μ'*	Virus excretion coefficient of the asymptomatic people	——	virus^−1^·mL^-3^·day^−1^ ·km^2^·person^−1^	*c*/*μ'*
*C*	Relative viral excretion ratio of asymptomatic individuals versus the symptomatic		1	Model fitting
*ɛ*	Virus’s extinction speed in water	0.1	day^-1^	Literature [27–35]
*Q*	Isolation coefficient	0–1	1	Actual
*E*	Infection probability when exposure to the virus once		1	Formula calculation
*M*	The average number of people a person can reach per day	15	Person	Literature [36]
*H*	Coefficient of the average number of people per person per day after school-closure	0–1	1	Assumption
*X*	Coefficient of infection probability when exposure to the virus once after disinfection	0–1	1	Assumption

When calculating *e,* if *β* ≥ 1, use the formula *e* = *β*/*m*; If *β*<1, use the formula *e* = 1 − (1 − *β*) ^1/^*^m^*.

*β*, *β_W_* and *c* were obtained by fitting the actual data with the model, and the time of peak incidence of each actual norovirus outbreak was used as a node and the data before the node were used to fit each outbreak to obtain the *β* and *β_W_* without interventions.

Studies have shown that the incubation period for norovirus infectious diarrhea is 12–48 h [17,18], with an average duration of 1–3 d [22–26], after the infection of norovirus, the time to begin excreting the virus is 36 h on average [19], with the excretion time can last for about 26 d [19], and the survival time of the norovirus in the external environment is about 7 to 12 d [27–35]. norovirus infectious diarrhea has a 30% share of asymptomatic [19–21], the asymptomatic individuals can also excrete the virus into the outside environment, and asymptomatic individuals are 0–25% as infectious as symptomatic individuals [16]. In addition, studies have shown that a person can contact an average of 15 people a day [36]. In this study, the incubation period was 1 d [17,18], the latent period was 1 d [19], the symptom duration was 3 d [22–26], the infectious period was 26 d [19], the proportion of asymptomatic people was 30% [19–21], the transmissibility rate of asymptomatic people relative to symptomatic people was 5% [16], the survival time of the virus in the external environment was 10 d [27–35], the average contact of a person with 15 people per day [36] was used to model this study, and we assumed that everyone who might come into contact in the school is homogeneous and mixed . If isolation measure is taken, this study assumes that cases will be sent to isolation within half a day after they appear. That is, in the model, *ω* = 1, *ω′* = 1, *p* = 0.3, *γ* = 1/3 = 0.3333, *γ′* = 1/26 = 0.03846, *γ″* = 1/0.5 = 2, *k* = 0.05, *ɛ* = 1/10 = 0.1, *m* = 15.

In this study, the implementation intensity of isolation, school-closure and disinfection measures were related to the assumed parameters *q* (isolation coefficient), *h* (coefficient of the average number of people per person per day after school-closure), and *x* (coefficient of infection probability when exposure to the virus once after disinfection). The value ranges of *q*, *h*, and *x* are all 0–1, and the interval between values is 0.1.

### Indicators for assessing transmissibility and control effectiveness

The transmissibility of norovirus can be quantified by the effective reproduction number *R_eff_*, which is the average number of symptomatic people that a symptomatic person can cause during its infection period. In the model, the calculation formula for *R_eff_* can be simplified to:

$$R_{eff}=\beta S\left(\frac{1-p}{\gamma }+\frac{kp}{\gamma^{\prime }}\right)+\beta_{w}S[\frac{\mu \left(1-p\right)}{\gamma \text{​}\varepsilon }+\frac{\mu \prime p}{\gamma^{\prime }\text{​}\varepsilon }]$$

In this study, the effect of various prevention and control measures was evaluated by the total attack rate (TAR) and the peak incidence (PI), the peak incidence in this study was the maximum incidence calculated in days. The cumulative number of symptomatic people (*n*) and the maximum number of new symptomatic people per day (n_p_) under various prevention and control measures of each outbreak can be obtained by model simulation, combined with the total number of exposed population (*N*), the total attack rate and the peak incidence can be calculated. The calculation formula is as follows:

$$\mathrm{TAR}=\frac{n\;}{N}\times 100\%$$

$$PI=\frac{n_{p}\;}{N}\times 100\%$$

### Sensitivity analysis

Since the parameters *ω*, *ω′*, *p*, *γ*, *γ′*, *k*, *ɛ* were obtained through literatures, sensitivity analysis for each parameter was performed by taking 10 values uniformly within its range.

### Model simulation and data processing methods

This study, EXCEL2020 was used for data entry and collecting. Statistical analysis of related data was performed on SPSS 26.0, MATLAB R2022a was used to simulate the model and visualize, the parameter output of the model fitting was determined by the criteria of least root mean square (LRMS). The ordinary differential equations were solved using the Runge–Kutta discretization scheme with adaptive step-size selection.

## Results

### Epidemiological characteristics

**Figure 2.**
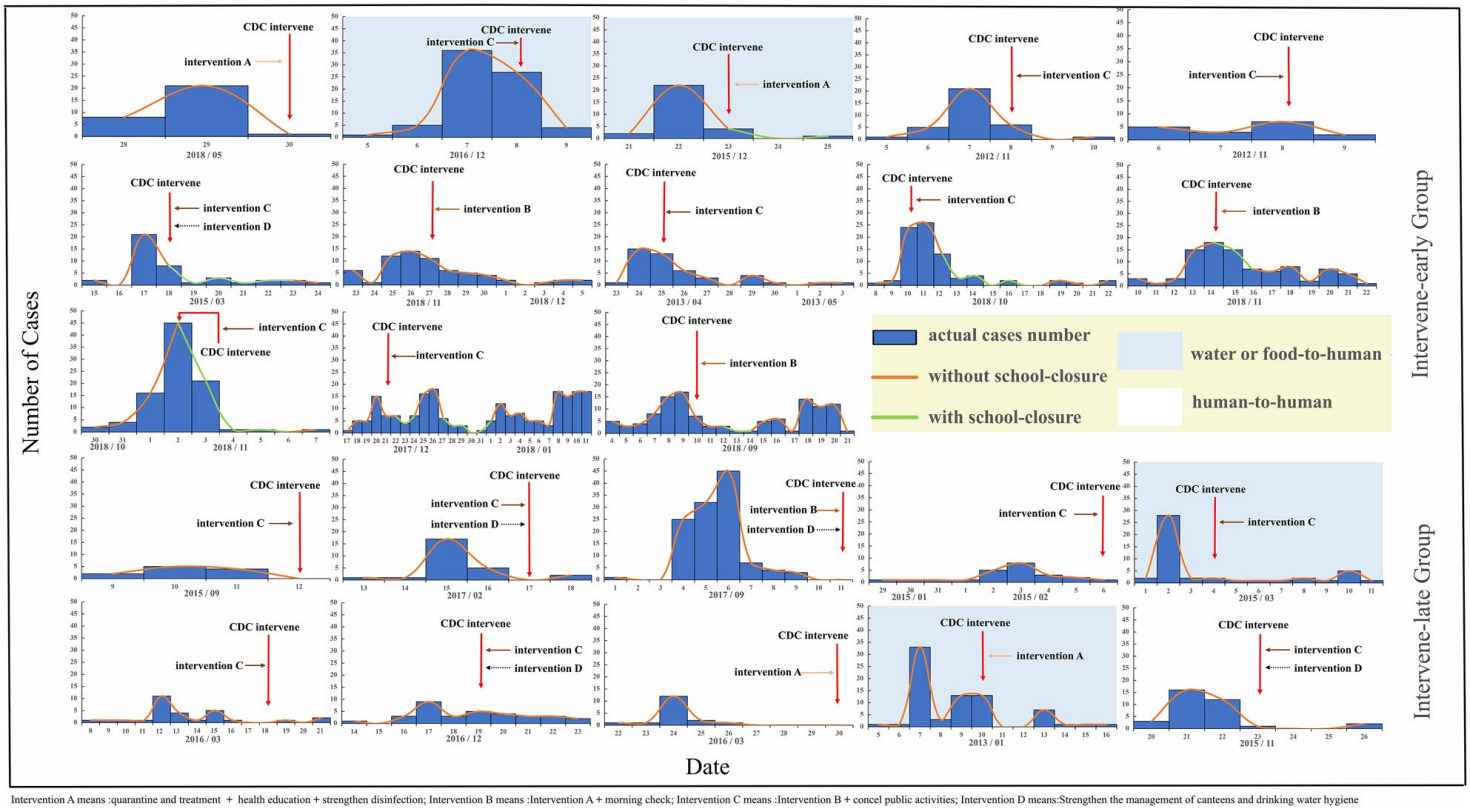
Epidemiological characteristics of 23 norovirus outbreaks. Intervention A means: quarantine and treatment + health education + strengthen disinfection; Intervention B means: Intervention A + morning check; Intervention C means: Intervention B + cancel public activities; Intervention D means: Strengthen the management of canteens and drinking water hygiene.

We obtained epidemiological investigation reports of 23 norovirus outbreaks and categorized these outbreaks into an early intervention group and a late intervention group according to whether the CDC intervened the day after the peak of the norovirus outbreak. 23 diarrheal outbreaks of norovirus infection and CDC interventions are shown below (Figure 2). The difference in the rate of norovirus transmission between the two groups was not statistically significant (Table 2).

**Table 2.** Comparison about the transmissibility of norovirus outbreaks in two groups (M, P25%–P75%).

Group	Number of outbreaks	Effective reproduction number (*R_eff_*)		*U*-value	*p*-value
Intervene-early	13	3.85	2.70,12.18	68.000	0.852
Intervene-late	10	6.73	3.25,9.43		

The effective reproduction number *R*_eff_ of the two groups was compared with Mann-Whitney *U* test.

### Analysis for the transmissibility of norovirus

For the 23 norovirus outbreaks in school, the average effective reproduction number in the human-to-human route was 8.92, with a 95% confidence interval (5.48, 12.37), and the average effective reproduction number in the water or food-to-human route was 2.19, and the 95% confidence interval was (1.14, 3.24).

**Figure 3.**
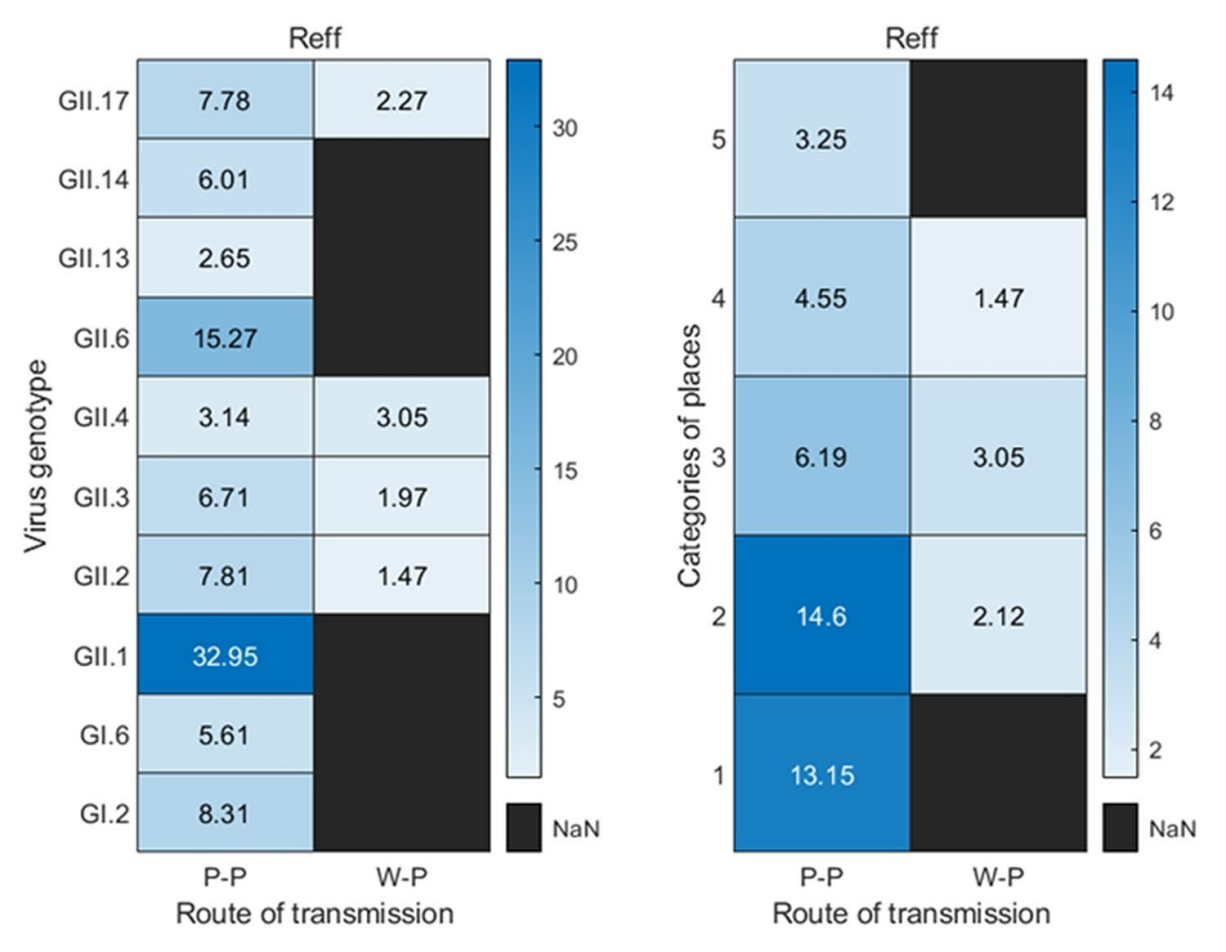
Calculation of *R_eff_* for various virus genotypes or places under two transmission routes. P-P means human-to-human transmission route, W-P means water or food -to-human transmission route, and 1–5 in categories of places mean kindergarten, primary school, middle school, Common Colleges and Secondary vocational school, and nine-year school and twelve-year school, respectively.

For the 19 norovirus outbreaks of the human-to-human route, including 10 virus genotypes, the *R_eff_* of GII.4 genotype (1 outbreak) and GII.13 (1 outbreak) were 3.14 and 2.65, respectively, both of which were lower than the 95% confidence interval of the *R_eff_* in the human-to-human transmission route; the average *R_eff_* of GII.6 (2 outbreaks) was 15.27, it was higher than the 95% confidence interval of the *R_eff_* in the human-to-human transmission route; and the *R_eff_* of the GII.1 subtype (1 outbreak) was 32.95, which was much higher than the 95% confidence interval of the *R_eff_* in the human-to-human transmission route. The average *R_eff_* of the 19 outbreaks in different sites was calculated, which showed that the effective reproduction number of norovirus outbreaks in kindergarten, primary school, middle school, Common Colleges and Secondary vocational school, and nine-year school and twelve-year school were 13.15 (2 outbreaks), 14.6 (6 outbreaks), 6.19 (5 outbreaks), 4.55 (4 outbreaks) and 3.25 (2 outbreaks), respectively. The effective reproduction number of the four norovirus outbreaks in the water or food-to-human route were all within the 95% confidence interval (Figure 3).

### Analysis for the effectiveness of prevention and control measures

**Figure 4.**
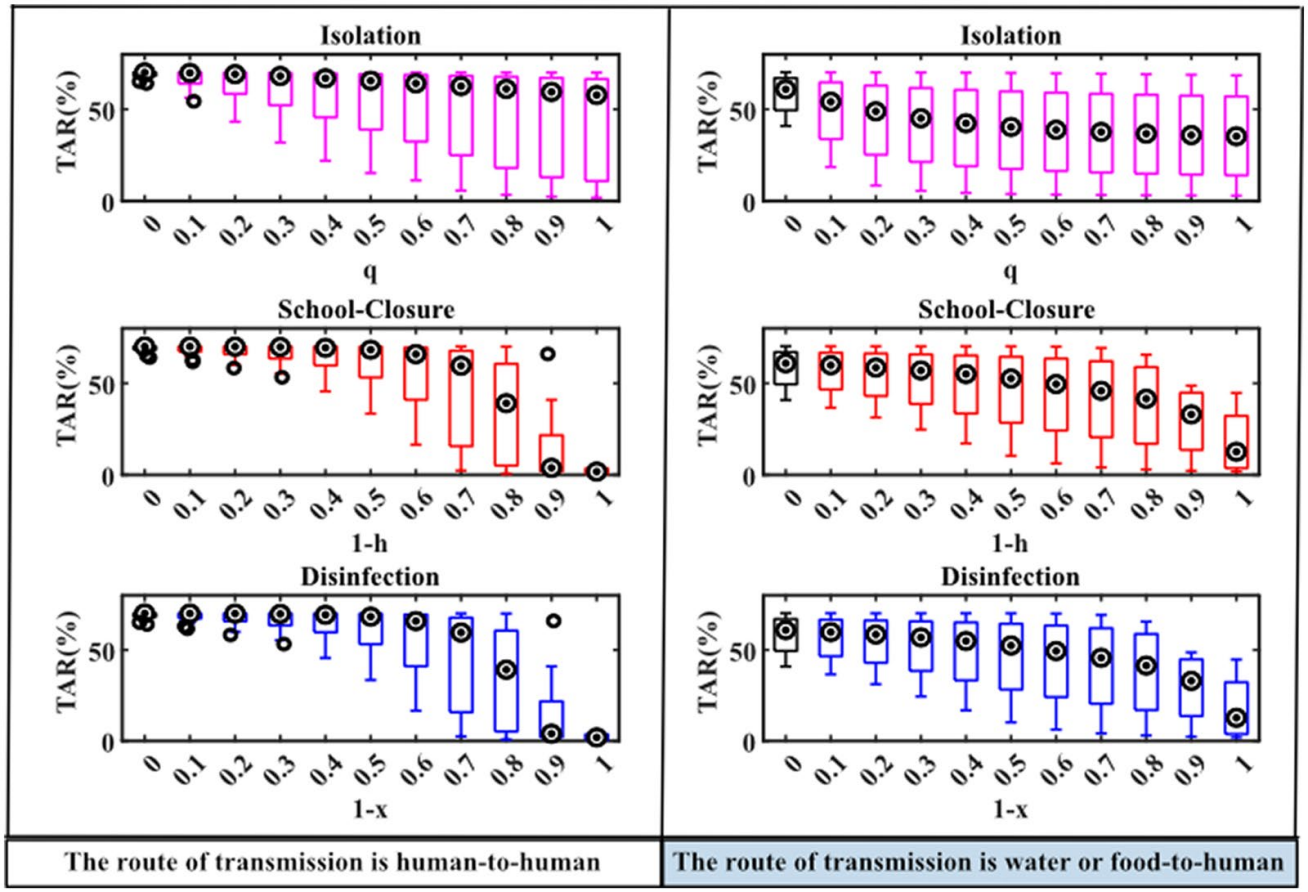
TAR simulation results under different interventions in two transmission routes. When the abscissa is 0, it means that there is no intervention. Details of these three parameters are shown in Table 1.

The total attack rates of norovirus outbreaks in schools without intervention, or with separate isolation, school-closure or disinfection measures under different intervention intensities were simulated as follows: The median (M) and interquartile range (IQR) of the total attack rate without intervention in the human-to-human route was 70.11 (68.24, 70.15)%, the total attack rate for a few outbreaks (7/19) decreased significantly with increasing isolation coefficients after the implementation of isolation measures, but the total attack rate obtained by other norovirus outbreaks simulations did not decrease significantly. And when the isolation coefficient was 1, the M (IQR) of the total attack rate from the 19 norovirus outbreaks simulations was 57.78 (8.69, 66.72)%. When the daily exposure coefficient (*h*) of the person was 0.1 or 0 after the school closure measures were implemented, the total attack rate obtained for each outbreak simulation was significantly reduced, with values of M (IQR) were 3.87 (1.80, 22.07)% and 1.82 (0.56, 3.66)%, respectively (Figure 4).

In the water or food-to-human route, the M (IQR) of the total attack rate without intervention was 60.92 (45.17, 68.61)%, and when isolation measures were implemented, the total attack rate for each epidemic gradually decreased as the isolation rate coefficient increased, and when the isolation coefficient was 1, the M (IQR) of the total attack rate was 35.28 (8.38, 62.74)%. When school closure measures were implemented, the total attack rate simulated with the same variation in person-to-person pathways, the values of M (IQR) were 33.02 (7.95, 46.78)% and 12.65 (2.73, 38.47)% when *h* was 0.1 or 0, respectively. There was no significant difference in the total attack rate of the two transmission routes simulated by school closures and disinfection measures at different intervention intensities (Figure 4).

**Figure 5.**
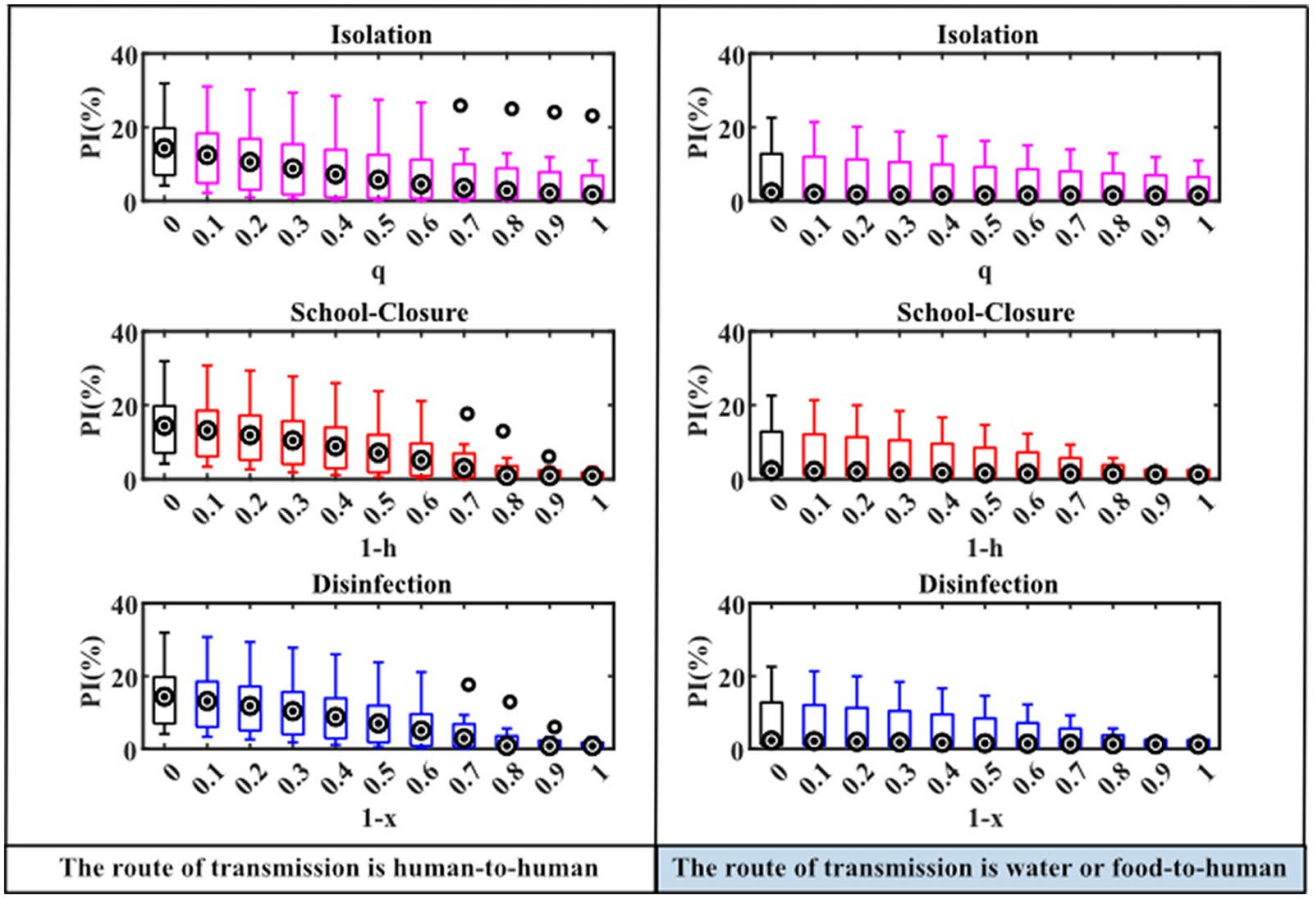
PI simulation results under different interventions in two transmission routes. When the abscissa is 0, it means that there is no intervention. Details of these three parameters are shown in Table 1.

The simulated peak incidence was: The M (IQR) of the peak incidence for the person-to-person route without intervention was 14.37 (7.01, 19.95)%. The simulation results for each outbreak with all three interventions showed that the peak incidence tended to decrease gradually by increasing intervention intensity. If taken isolation intervention, the M (IQR) of the peak incidence was 1.73 (0.57, 7.05)% when the isolation coefficient was 1. When school closure measures were implemented and *h* was 0.3, 0.2, 0.1 or 0, respectively, the M (IQR) of the peak incidence of each epidemic simulation decreased significantly to 2.94 (0.54, 7.01)%, 0.91 (0.54, 3.68)%, 0.86 (0.28, 2.49)% and 0.86 (0.28, 2.04)% (Figure 5).

In the water or food-to-human pathway, the M (IQR) of the peak incidence was 2.36 (0.87, 17.69) % without interventions, and the change in peak incidence of outbreaks after the implementation of interventions was the same as that in the human-to-human route. When isolation measures were implemented and the isolation coefficient was 1, the M (IQR) of the peak incidence was 1.44 (0.31, 8.74)%. When school closure measures were implemented and *h* was 0.3, 0.2, 0.1 or 0, respectively, the M (IQR) of the simulated peak incidence were 1.44 (0.31, 7.45)%, 1.37 (0.30, 4.73), 1.32 (0.29, 3.00)%, and 1.27 (0.28, 2.94)%. There were no significant differences in peak incidence simulated between school closure and disinfection measures at different intervention intensities under both transmission routes (Figure 5).

### Sensitivity analysis

**Figure 6.**
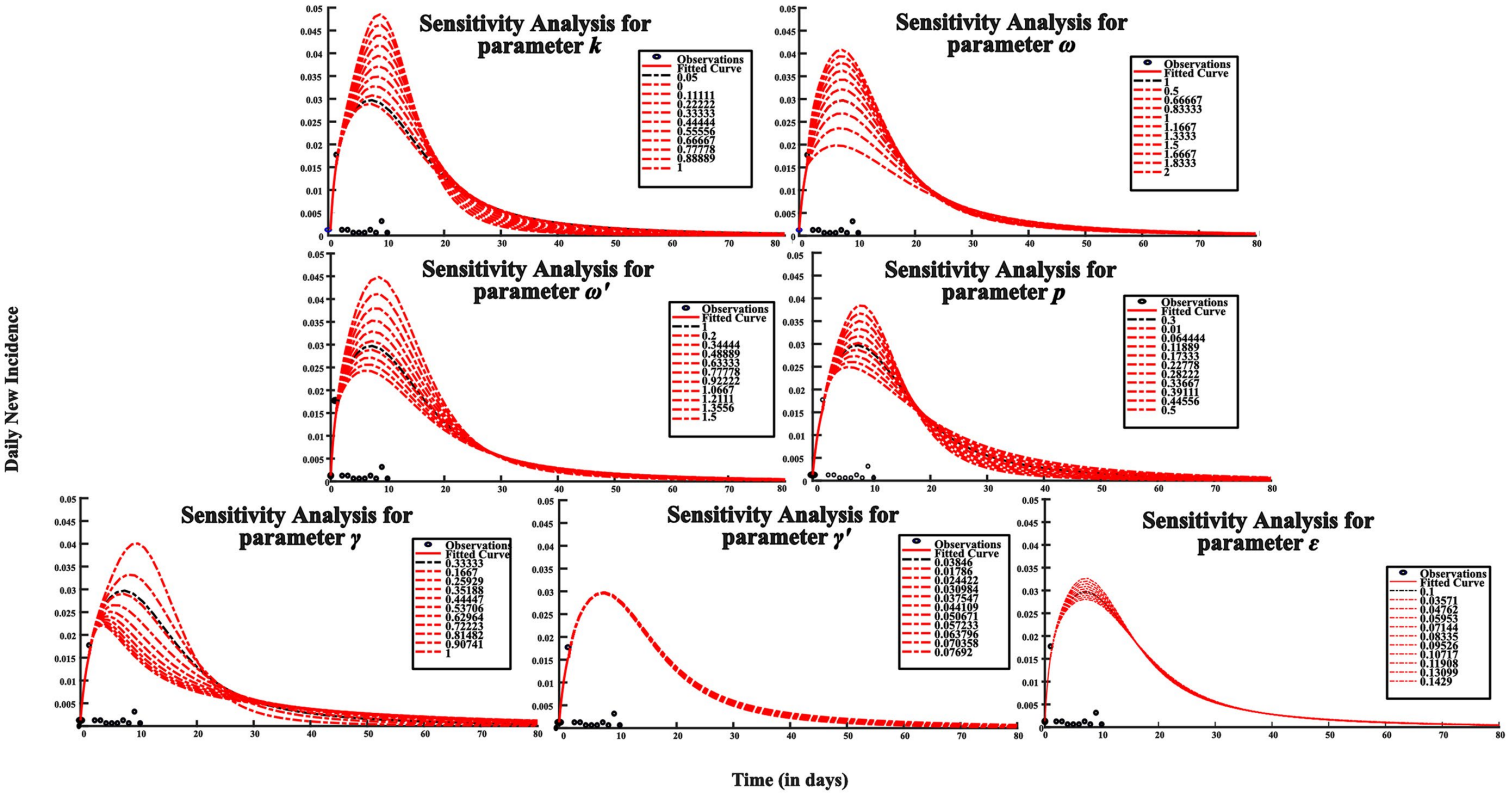
Sensitivity analysis results of SEAIQRW model.

When values of the parameters k, ω, p, γ, γ′, and ɛ are varied, simulation results vary significantly, but there was no obvious difference in the simulation results when varying parameter *ω*′ (Figure 6). It has proven that the model had certain sensitivity to the parameters *k, ω, p, γ, γ*′*, ɛ,* while the parameter *ω*′ was less sensitive in this model.

## Discussion

In this study, all the norovirus outbreaks chosen in water or food to human route were GII genomes, which may validates the finding that noroviruses of GII genomes are more likely to be transmitted through water than GI genomes [37], and it is also consistent with the results of surveillance of noroviruses with predominantly GII genomes in sewage from another province (Guangdong Province) in China [38]. The model simulation data without intervention in this study showed that norovirus outbreaks in school premises under both transmission routes could lead to symptoms in about 2/3 of the population. This is consistent with the extreme susceptibility of norovirus to cause transmission and suggests the importance of proposing effective prevention and control measures for norovirus outbreaks.

When calculating the effective reproduction number (*R_eff_*) in school under different transmission routes, each outbreak among people had a high average effective reproduction number (8.92), and the effective reproduction number of outbreaks was much higher in kindergarten or primary school than that in the total population [16], which may result from the high density of people in schools, especially kindergartens or primary schools, where have the high number of gathering activities, the high exposure, and the poor hygiene habits of young children. However, the mean effective reproduction number in the water or food-to-person route was smaller than that in the person-to-person route and was not significantly different from the total population. The results of the *R_eff_* were close to those calculated for norovirus outbreaks *(R_0_^direct^* = 5.9, *R_0_^environment^* = 1.6) on cruise ships where the population is also of density and contacts frequently [39]. Besides, the effective reproduction number of 1 GII.1 genotype norovirus outbreak and 2 GII.6 genotype norovirus outbreaks were much higher than the general population. This may indicate that the genotypes of these two noroviruses are more likely to cause transmission; However, on the other hand, there are many factors that affect the effective reproduction number, such as population aggregation, behavioral patterns, exposure to infectious agents, and climatic conditions, so the effective reproduction number may be overestimated due to the small number of outbreaks when calculating the effective reproduction number.

The simulated results of isolation show that it can effectively reduce the peak incidence, the larger the isolation rate coefficient, the better the effect. But it is less effective in preventing and controlling the total attack rate of the outbreak, and when the isolation rate coefficient is 1, the total attack rate of each outbreak in both transmission routes is reduced by less than 50%, which is consistent with the finding of isolation implementation [40]. The reason for this analysis may be that isolation measures only target symptomatic individuals in an outbreak, but after norovirus transmission, up to 30% of asymptomatic individuals are infected and they are also contagious and the outbreak can continue to spread.

The results of the epidemic prevention and control effect when the school closure measures were taken showed that with the increase of intensity, when the effect of school closure reached more than 90% (*h* is less than or equal to 0.1), the total attack rate and the peak incidence of each outbreak were significantly lower than those when there was no intervention, which indicated that the timely adoption of large-scale school closure measures after a norovirus outbreak could effectively reduce the scale of the outbreak, which was in line with the results of previous studies [12,13]. This study focused only on epidemiological indicators of total attack rate and peak incidence, but the impact of economic, psychological, social, and educational stability factors should also be considered in the actual implementation of school-closure measures.

When disinfection measures were taken, there was no significant difference from school-closure measures, that is, when the disinfection effect reached 90% or more (*x* is less than or equal to 0.1), the disinfection measures could effectively control the transmission of norovirus, which was consistent with the research results of Chen Tianmu [13]. Therefore, it can be concluded that standardized disinfection of the external environment immediately after an outbreak in schools is a key measure to prevent and control the outbreak. Therefore, the following prevention and control recommendations are made for norovirus outbreaks in schools: after prompt isolation measures for all symptomatic cases, mass school closures or standardized disinfection of the external environment should be facilitated immediately.

Outbreaks of norovirus in Jiangsu Province have persisted in recent years [41,42], but the research on norovirus in Jiangsu Province has mostly focused on the classification of viruses [15,41,43], and there is a lack of comprehensive evaluation studies on specific interventions in specific scenarios. In this study, based on our previous studies, we constructed intervention models of isolation, school closure and disinfection measures for 23 norovirus outbreaks in Jiangsu Province based on the SEIAQRW model, and evaluated the effects of various interventions for norovirus outbreaks by setting different intervention intensities, which made up for the lack of research in Jiangsu Province only for epidemiological characteristics in recent years, and also enriched the research content of mathematical models in the field of norovirus research. These reflect the innovative significance of this study. In addition, the subjects selected for this study included multiple virus genotypes, different transmission routes, and multiple outbreak sites, so the results are more extrapolative than previous studies.

## Limitations

The present study still has shortcomings. First, limited by model fitting, some parameters relied on literature and some relied on model fitting, but some of the outbreak data selected in this study were small, so there may be uncertainty in the fitted parameters, and the remaining parameters relied on literature, which may have an impact on the certainty and accuracy of the study. Second, the transmissibility analysis of the 23 norovirus outbreaks in this study was based on the timing of the CDC intervention, showing no significant difference between the early and late intervention groups. But norovirus infections with diarrhea usually have a short duration of illness at the time of the actual outbreak, schools or parents generally take control measures such as treatment and home rest for symptomatic students earlier than CDC interventions; therefore, the number of norovirus outbreaks and the accuracy of outbreak investigations should be improved in follow-up studies to verify whether or how the timing of interventions affects the transmissibility of norovirus. Finally, this study only evaluated the prevention and control effects of some key individual interventions for norovirus outbreaks; other interventions, or the combined prevention and control effects of various interventions, have not been studied.

## Conclusion

Calculations for *R_eff_* show that norovirus is easily transmitted in schools especially through the human-to-human route, and it could eventually lead to more than two-thirds of the population becoming infected when interventions were absent. Norovirus outbreaks’ prevention and control measures in schools, such as school-closure or the disinfection effect greater than 90%, can effectively reduce the total attack rate (TAR) and peak incidence (PI) of a norovirus outbreak, and the implementation of isolation measures only has a good effect on the reduction of the peak incidence, but the three measures alone may not completely prevent the spread of norovirus. To better control the spread of norovirus in school premises, other prevention and control measures, such as widespread school closures or standardized disinfection, should be supplemented by timely isolation of all cases.

## Data Availability

All data and associated code can be found on GitHub (https://github.com/WangJing2478/NorovirusJiangsu).

## References

[1] Ahmed SM, Hall AJ, Robinson AE, et al. Global prevalence of norovirus in cases of gastroenteritis: a systematic review and meta-analysis. Lancet Infect Dis. 2014;14(8):1–12. doi: 10.1016/S1473-3099(14)70767-4. 24981041 PMC8006533

[2] Nguyen GT, Phan K, Teng I, et al. A systematic review and meta-analysis of the prevalence of norovirus in cases of gastroenteritis in developing countries. Medicine. 2017;96(40):e8139. doi: 10.1097/MD.0000000000008139. 28984764 PMC5738000

[3] Hall AJ, Vinjé J, Lopman B, et al. Updated norovirus outbreak management and disease prevention guidelines. MMWR Recomm Rep. 2011;60(RR-3):1–18. 21368741

[4] Morillo SG, Timenetsky MCST Norovirus: an overview. Rev Assoc Med Bras. 2011;57(4):453–458. doi: 10.1590/s0104-42302011000400023. 21876931

[5] Riddle MS, Walker RI. Status of vaccine research and development for norovirus. Vaccine. 2016;34(26):2895–2899. doi: 10.1016/j.vaccine.2016.03.077. 27036510

[6] Teunis PF, Moe CL, Liu P, et al. Norwalk virus: how infectious is it? J Med Virol. 2008;80(8):1468–1476. doi: 10.1002/jmv.21237. 18551613

[7] Banyai K, Estes MK, Martella V, et al. Viral gastroenteritis. Lancet. 2018;392(10142):175–186. doi: 10.1016/S0140-6736(18)31128-0. 30025810 PMC8883799

[8] Yezli S, Otter JA. Minimum infective dose of the major human respiratory and enteric viruses transmitted through food and the environment. Food Environ Virol. 2011;3(1):1–30. doi: 10.1007/s12560-011-9056-7. 35255645 PMC7090536

[9] Qin SW, Chan TC, Cai J, et al. Genotypic and epidemiological trends of acute gastroenteritis associated with noroviruses in China from 2006 to 2016. Int J Environ Res Public Health. 2017;14(11):1341. doi: 10.3390/ijerph14111341. 29099784 PMC5707980

[10] Jin M, Wu S, Kong X, et al. Norovirus outbreak surveillance, China, 2016-2018. Emerg Infect Dis. 2020;26(3):437–445. doi: 10.3201/eid2603.191183. 32091361 PMC7045832

[11] Yu F, Jiang B, Guo X, et al. Norovirus outbreaks in China, 2000-2018: a systematic review. Rev Med Virol. 2022;32(6):e2382. doi: 10.1002/rmv.2382. 35946340

[12] Xu Y, Zhang R, Zhou Z, et al. Assessment on transmission capacity of norovirus infection and effect of control measures in schools. Chin J Public Health. 2021;37(4):702. doi: 10.11847/zgggws1131517.

[13] Yu G, Chen T, Zhu Y, et al. Effect of prevention and control measures of a norovirus infectious outbreak based on dynamic model disease surveillance. 2021;36(12):1312–1318. doi: 10.3784/jbjc.202103040101.

[14] Xu Y, Zhu Y, Lei Z, et al. Investigation and analysis on an outbreak of norovirus infection in a health school in Guangdong province, China. Infect Genet Evol. 2021;96:105135. doi: 10.1016/j.meegid.2021.105135. 34781036

[15] Ai J, Zhu Y, Fu J, et al. Study of risk factors for total attack rate and transmission dynamics of norovirus outbreaks, Jiangsu province, China, from 2012 to 2018. Front Med. 2021;8:786096. doi: 10.3389/fmed.2021.786096. PMC8777030 35071268

[16] Gaythorpe KAM, Trotter CL, Lopman B, et al. Norovirus transmission dynamics: a modelling review. Epidemiol Infect. 2018;146(2):147–158. doi: 10.1017/S0950268817002692. 29268812 PMC5851036

[17] Hutson AM, Atmar RL, Estes MK. Norovirus disease: changing epidemiology and host susceptibility factors. Trends Microbiol. 2004;12(6):279–287. doi: 10.1016/j.tim.2004.04.005. 15165606 PMC7172956

[18] Caul EO. Small round structured viruses: airborne transmission and hospital control. Lancet. 1994;343(8908):1240–1242. doi: 10.1016/s0140-6736(94)92146-6. 7910270

[19] Atmar RL, Opekun AR, Gilger MA, et al. Norwalk virus shedding after experimental human infection. Emerg Infect Dis. 2008;14(10):1553–1557. doi: 10.3201/eid1410.080117. 18826818 PMC2609865

[20] Graham DY, Jiang X, Tanaka T, et al. Norwalk virus infection of volunteers: new insights based on improved assays. J Infect Dis. 1994;170(1):34–43. doi: 10.1093/infdis/170.1.34. 8014518

[21] Phillips G, Lopman B, Tam CC, et al. Diagnosing norovirus-associated infectious intestinal disease using viral load. BMC Infect Dis. 2009;9:63. doi: 10.1186/1471-2334-9-63. 19442278 PMC2698835

[22] Atmar RL, Estes MK. The epidemiologic and clinical importance of norovirus infection. Gastroenterol Clin North Am. 2006;35(2):275–290, viii. doi: 10.1016/j.gtc.2006.03.001. 16880066

[23] Kaplan JE, Schonberger LB, Varano G, et al. An outbreak of acute nonbacterial gastroenteritis in a nursing home. Demonstration of person-to-person transmission by temporal clustering of cases. Am J Epidemiol. 1982;116(6):940–948. doi: 10.1093/oxfordjournals.aje.a113496. 6293306

[24] Rockx B, De Wit M, Vennema H, et al. Natural history of human calicivirus infection: a prospective cohort study. Clin Infect Dis. 2002;35(3):246–253. doi: 10.1086/341408. 12115089

[25] Lopman BA, Reacher MH, Vipond IB, et al. Clinical manifestation of norovirus gastroenteritis in health care settings. Clin Infect Dis. 2004;39(3):318–324. doi: 10.1086/421948. 15306997

[26] Kaplan JE, Feldman R, Campbell DS, et al. The frequency of a norwalk-like pattern of illness in outbreaks of acute gastroenteritis. Am J Public Health. 1982;72(12):1329–1332. doi: 10.2105/ajph.72.12.1329. 6291414 PMC1650540

[27] Dalling J. A review of environmental contamination during outbreaks of norwalk-like virus. British Journal of Infection Control. 2004;5(2):9–13. doi: 10.1177/14690446040050020301.

[28] Mattison K, Karthikeyan K, Abebe M, et al. Survival of calicivirus in foods and on surfaces: experiments with feline calicivirus as a surrogate for norovirus. J Food Prot. 2007;70(2):500–503. doi: 10.4315/0362-028x-70.2.500. 17340890

[29] D'Souza DH, Sair A, Williams K, et al. Persistence of caliciviruses on environmental surfaces and their transfer to food. Int J Food Microbiol. 2006;108(1):84–91. doi: 10.1016/j.ijfoodmicro.2005.10.024. 16473426

[30] Wu HM, Fornek M, Schwab KJ, et al. A norovirus outbreak at a long-term-care facility: the role of environmental surface contamination. Infect Control Hosp Epidemiol. 2005;26(10):802–810. doi: 10.1086/502497. 16276954

[31] Jones EL, Kramer A, Gaither M, et al. Role of fomite contamination during an outbreak of norovirus on houseboats. Int J Environ Health Res. 2007;17(2):123–131. doi: 10.1080/09603120701219394. 17616868

[32] Gallimore CI, Taylor C, Gennery AR, et al. Environmental monitoring for gastroenteric viruses in a pediatric primary immunodeficiency unit. J Clin Microbiol. 2006;44(2):395–399. doi: 10.1128/JCM.44.2.395-399.2006. 16455890 PMC1392667

[33] Kuusi M, Nuorti JP, Maunula L, et al. A prolonged outbreak of norwalk-like calicivirus (NLV) gastroenteritis in a rehabilitation Centre due to environmental contamination. Epidemiol Infect. 2002;129(1):133–138. doi: 10.1017/s0950268802007276. 12211580 PMC2869858

[34] Cheesbrough JS, Green J, Gallimore CI, et al. Widespread environmental contamination with norwalk-like viruses (NLV) detected in a prolonged hotel outbreak of gastroenteritis. Epidemiol Infect. 2000;125(1):93–98. doi: 10.1017/s095026889900432x. 11057964 PMC2869574

[35] Green J, Wright PA, Gallimore CI, et al. The role of environmental contamination with small round structured viruses in a hospital outbreak investigated by reverse-transcriptase polymerase chain reaction assay. J Hosp Infect. 1998;39(1):39–45. doi: 10.1016/s0195-6701(98)90241-9. 9617683

[36] Zhang J, Litvinova M, Liang Y, et al. Changes in contact patterns shape the dynamics of the COVID-19 outbreak in China. Science. 2020;368(6498):1481–1486. doi: 10.1126/science.abb8001. 32350060 PMC7199529

[37] Lysen M, Thorhagen M, Brytting M, et al. Genetic diversity among food-borne and waterborne norovirus strains causing outbreaks in Sweden. J Clin Microbiol. 2009;47(8):2411–2418. doi: 10.1128/JCM.02168-08. 19494060 PMC2725682

[38] Lu J, Peng J, Fang L, et al. Capturing noroviruses cir­culating in the population: sewage surveillance in Guangdong, China (2013–2018). Water Res. 2021;196:116990. doi: 10.1016/j.watres.2021.116990. 33725645

[39] Towers S, Chen J, Cruz C, et al. Quantifying the relative effects of environmental and direct transmission of norovirus. R Soc Open Sci. 2018;5(3):170602. doi: 10.1098/rsos.170602. 29657742 PMC5882666

[40] Chen T, Gu H, Leung RK, et al. Evidence-based interventions of norovirus outbreaks in China. BMC Public Health. 2016;16(1):1072. doi: 10.1186/s12889-016-3716-3. 27729034 PMC5059926

[41] Fu J, Bao C, Huo X, et al. Increasing recombinant strains emerged in norovirus outbreaks in Jiangsu, China: 2015–2018. Sci Rep. 2019;9(1):20012. doi: 10.1038/s41598-019-56544-2. 31882797 PMC6934623

[42] Fu JG, Shi C, Xu C, et al. Outbreaks of acute gastroenteritis associated with a re-emerging GII.P16-GII.2 norovirus in the spring of 2017 in Jiangsu, China. PLOS One. 2017;12(12):e0186090. doi: 10.1371/journal.pone.0186090. 29284004 PMC5746213

[43] Fang X, Ai J, Liu W, et al. Epidemiology of infectious diarrhoea and the relationship with etiological and meteorological factors in Jiangsu province, China. Sci Rep. 2019;9(1):19571. doi: 10.1038/s41598-019-56207-2. 31862956 PMC6925108

